# Fluid Metabolism in Athletes Running Seven Marathons in Seven Consecutive Days

**DOI:** 10.3389/fphys.2018.00091

**Published:** 2018-02-12

**Authors:** Daniela Chlíbková, Pantelis T. Nikolaidis, Thomas Rosemann, Beat Knechtle, Josef Bednář

**Affiliations:** ^1^Centre of Sports Activities, Brno University of Technology, Brno, Czechia; ^2^Exercise Physiology Laboratory, Nikaia, Greece; ^3^Institute of Primary Care, University of Zurich, Zurich, Switzerland; ^4^Medbase St. Gallen Am Vadianplatz, St. Gallen, Switzerland; ^5^Faculty of Mechanical Engineering, Brno University of Technology, Brno, Czechia

**Keywords:** multi-stage marathon, endurance, running, hydration status, sodium

## Abstract

**Purpose:** Hypohydration and hyperhydration are significant disorders of fluid metabolism in endurance performance; however, little relevant data exist regarding multi-stage endurance activities. The aim of the present study was to examine the effect of running seven marathons in 7 consecutive days on selected anthropometric, hematological and biochemical characteristics with an emphasis on hydration status.

**Methods:** Participants included 6 women and 20 men (age 42.6 ± 6.2 years). Data was collected before day 1 (B_1_) and after day 1 (A_1_), 4 (A_4_), and 7 (A_7_).

**Results:** The average marathon race time was 4:44 h:min (ranging from 3:09 – 6:19 h:min). Plasma sodium, plasma potassium and urine sodium were maintained during the race. Body mass (*p* < 0.001, η^2^ = 0.501), body fat (*p* < 0.001, η^2^ = 0.572) and hematocrit (*p* < 0.001, η^2^ = 0.358) decreased. Plasma osmolality (Posm) (*p* < 0.001, η^2^ = 0.416), urine osmolality (Uosm) (*p* < 0.001, η^2^ = 0.465), urine potassium (*p* < 0.001, η^2^ = 0.507), urine specific gravity (Usg) (*p* < 0.001, η^2^ = 0.540), plasma urea (PUN) (*p* < 0.001, η^2^ = 0.586), urine urea (UUN) (*p* < 0.001, η^2^ = 0.532) and transtubular potassium gradient (*p* < 0.001, η^2^ = 0.560) increased at A_1_, A_4_, and A_7_ vs. B_1_. Posm correlated with PUN at A_1_ (*r* = 0.59, *p* = 0.001) and A_4_ (*r* = 0.58, *p* = 0.002). The reported post-race fluid intake was 0.5 ± 0.2 L/h and it correlated negatively with plasma [Na^+^] (*r* = −0.42, *p* = 0.007) at A_4_ and (*r* = −0.50, *p* = 0.009) at A_7_. Uosm was associated with UUN at A_1_ (*r* = 0.80, *p* < 0.001), at A_4_ (*r* = 0.81, *p* < 0.001) and at A_7_ (*r* = 0.86, *p* < 0.001) and with Usg (*r* = 0.71, *p* < 0.001) at A_1_, (*r* = 0.52, *p* = 0.006) at A_4_ and (*r* = 0.46, *p* = 0.02) at A_7_.

**Conclusions:** Despite the decrease in body mass, fluid and electrolyte balance was maintained with no decrease in plasma volume after running seven marathons in seven consecutive days. Current findings support the hypothesis that body mass changes do not reflect changes in the hydration status during prolonged exercise.

## Introduction

Hypohydration and hyperhydration are significant disorders of fluid metabolism in endurance performance (Hew-Butler et al., 2015). Ultramarathon running races, generally performed on a variety of off-road terrains, are good models for the study of physiological responses to extreme stress (Millet and Millet, 2012), as they alter normal physiological processes and often result in fluid and electrolyte imbalance (Noakes et al., 2005).

Altered hydration status has not been well studied in multi-stage races (Krabak et al., 2017). In a multi-stage ultramarathon, athletes often compete for several days (Knechtle et al., 2012a), run at a slow pace (Costa et al., 2013), and are at an increased risk of fluid overload (Rüst et al., 2012). In case of excess fluid consumption, an increase in body mass, total body water and a decrease in plasma sodium [Na^+^] (Hew-Butler et al., 2015) can be expected. Exercise-associated hyponatremia (EAH) is a common occurrence among endurance athletes (Hew-Butler et al., 2015) and the incidence of EAH in multi-stage marathons is similar to its incidence in marathons and single-stage ultramarathons (Krabak et al., 2017). EAH occurred during multi-stage races with both increased and stable levels of total body water (Knechtle et al., 2011, 2012a; Rüst et al., 2012; Costa et al., 2013).

By contrast, anecdotal evidence suggests that multi-stage ultra-runners do not consume sufficient fluid to maintain euhydration, especially in hot ambient conditions (Costa et al., 2013). During multi-stage races, body mass often decreases (Knechtle et al., 2008a,b, 2010a,b, 2011, 2012b, 2015; Zouhal et al., 2009; Rüst et al., 2012; Costa et al., 2013; Hue et al., 2014; Krabak et al., 2017) or remains unchanged (Knechtle and Kohler, 2007; Knechtle et al., 2008c, 2010b). The decrease in body mass and the increase in urine specific gravity can indicate dehydration (Kavouras, 2002). Hypohydration from an athlete's perspective represents 2–5% body mass (volume) deficit (McDermott et al., 2017). Nevertheless, recent studies suggest that body mass loss for the maintenance of euhydration is 3%, or even 4%, depending on exercise duration (Noakes et al., 2005; Hoffman and Stuempfle, 2014, 2016), as body mass is ~1% higher at the race start than the day before (Hoffman et al., 2013a; Hoffman and Stuempfle, 2014). Therefore, it seems that body mass is not an accurate surrogate for body fluid volume in prolonged endurance races (Hew-Butler et al., 2015; Hoffman et al., 2017) and body mass losses of 1.9–5% are required in order to maintain euhydration during prolonged exercise (Hoffman et al., 2017). It is worth mentioning that that the appropriate body mass loss depends on the exercise duration. Multistage ultramarathon runners were most likely dehydrated after the last and longest stage of the race in a prospective observational cohort study (Krabak et al., 2017) though with the highest EAH prevalence. Contributing factors to body mass losses can be excessive distances, limited access to fluids and gastrointestinal problems (Hew-Butler et al., 2015).

In multi-stage races, plasma volume increases (Fellmann et al., 1999; Knechtle et al., 2010a, 2011, 2012a; Costa et al., 2013) or remains stable (Zouhal et al., 2009; Rüst et al., 2012). Despite an increase in plasma volume, plasma [Na^+^] (Wade et al., 1981; Knechtle et al., 2010a, 2011, 2012a, 2015; Rüst et al., 2012; Costa et al., 2013; Hue et al., 2014) and urinary [Na^+^] volumes (Knechtle et al., 2011, 2012a,b) often remain unchanged. The activity of aldosterone and antidiuretic hormone (ADH) is presumably the reason for maintained fluid homeostasis in athletes competing in multi-stage races (Knechtle et al., 2011, 2012a; Rüst et al., 2012).

To date, multi-stage races with distances longer or shorter than the marathon in at least one stage have been examined (Fellmann et al., 1999; Knechtle and Kohler, 2007; Knechtle et al., 2008a,c, 2010a,b, 2011, 2012a,b, 2015). However, we have not found any study investigating the effect of a multi-stage race—in which athletes are required to run one marathon per day for seven consecutive days—on the parameters of fluid metabolism using multiple hydration assessment techniques. Only one recent case study mentioned a recreational female runner who completed a multi-day endurance running event consisting of 26 marathon distances in 26 consecutive days (McManus et al., 2017) and the paper specifically focused on nutrition education. Although this case study has improved our understanding of fluid metabolism in ultra-endurance activities, there was only one subject, and thus, further research is needed. Recent studies (Maughan et al., 2007; Hoffman et al., 2017) suggest that changes in body mass do not reflect changes in the hydration status of those who participate in prolonged exercise. By contrast, hydration guidelines indicate that it is necessary to avoid body mass loss of more than 2% during exercise (American College of Sports Medicine et al., 2007). Therefore, the aim of the present study was to examine the effect of running seven marathons in seven consecutive days on selected anthropometric, hematological and biochemical characteristics with an emphasis on fluid metabolism. Based upon previous findings from studies investigating multi-stage ultramarathons, we hypothesized that body mass would decrease and our aim was then to examine if body mass changes while running marathons for several consecutive days reflect changes in the hydration status of the runners.

## Methods

The approval to conduct the study was obtained from the local institutional ethics committee at the Centre of Sports Activities, Brno University of Technology, Brno, Czechia. The athletes were encouraged to participate through links on the website. In addition, they were contacted via e-mail 3 months before the race and asked to participate in our investigation. Participation requirements for these competitions stipulate that the athletes have to be at least 18 years of age. No other inclusion/exclusion criteria were used, except that they finish the race. Thirty amateur ultra-runners who registered for the “Moravian Ultra Marathon” of 2015 volunteered to participate in the study. However, four of them did not complete all marathon races and were excluded from all analyses, resulting in a final sample of 26 runners (6 women and 20 men, age 42.6 ± 6.2 years). All volunteers provided written informed consent after the risks and benefits of the study were carefully explained to them.

### The race

The study was performed at the 2015 “Moravian Ultra Marathon,” an international running multi-stage race, which consists of seven marathons in seven consecutive days. It is considered the longest and most difficult multi-stage race in Czechia. The race was held in Lomnice from 28 June to 4 July 2015. For stage 1, all runners start at the same time—at 2:00 pm. Based on the results from stage 1, participants with a performance under 4:30 h start at 3:00 p.m. on the second day, while the others start at 2:00 p.m. For the last stage (stage 7), the runners have individual starting times, beginning at 8:00 and based on their average performance during the week (the previous results from each stage). The final time is measured individually, from the starting time of each participant. The winner is the athlete with the lowest total time from all stages. Stages are point-to-point distance, with a different route every day. Stages 1 and 7 started and finished in Lomnice, while stages 2, 3, 4, 5, and 6 each started at a different location. The racers were taken by bus prior to the start, and each stage finished in Lomnice. Each stage has the approximate length of a classical marathon (42.2–43.0 km) and runs through very rugged terrain, with an average ascent of 900 m, which makes it certainly more challenging than other races organized in Czechia. The route took place on an asphalt surface, through both light traffic and fields and woods. Table 1 shows ascents and descents and general weather conditions during all the stages of the “Moravian Ultra Marathon.” The temperature rose gradually from stage to stage, and increased by 12 degrees during stage 7. Each stage had six refreshment stations where food and beverages, such as water, sports drinks, tea, soup, caffeinated drinks, mineral water, fresh, and dry fruit, cheese, salty and sweet biscuits, salty crisps, peanuts and chocolate (until it melted in the heat), were available. Runners could also use their own refreshments.

**Table 1:** Stages of the “Moravian ultra marathon”: ascents, descents and general weather conditions.

**Stage**	**Ascent (m)**	**Descent (m)**	**General weather conditions**	**Temperature range (°C)**	**Humidity (%)**
1	830	830	Sun, wind	18–20	31.6
2	830	862	Sun, wind	18–20	48.2
3	830	734	Sun	24–25	36.2
4	900	787	Sun	25–26	97.3
5	760	948	Sun	28–30	66.4
6	911	1,055	Sun	25–26	37.9
7	740	740	Sun	28–30	44.1

### Measurements and calculations

Participants recorded the amount of fluids with which they started the race. Then, at each fluid station, there were assistants marking the number of cups consumed by the athletes. Fluids that were part of a meal or snack were not recorded. Immediately after each stage, during post-race measurements, the total fluid intake was estimated based on the data from the athletes and assistants. The athletes did not report solid food intake. We are aware of the fact that fluids from the fluid station could sometimes be used for other purposes than drinking (cooling, etc.). However, we assume that this was done predominantly near the fluid station, and the assistants were asked to mark the number of actually consumed cups. The organizer did not provide the athletes with any special advice on the website regarding what or how much they should drink during the race.

Blood and urine samples were collected before stage 1 (B_1_). Due to the demanding conditions of the 7-day race and limitations related to performing research at a competitive event, we decided to perform further sampling during the race only after stage 1 (A_1_), 4 (A_4_), and 7 (A_7_). Standardization of the sitting position prior to blood collection was respected. The measurement included a mid-flow urine sample prior to body mass measurements. Blood and urine samples were immediately transported to the laboratory and analyzed within 6 h. After venipuncture of the antecubital vein, two Sarstedt S-Monovettes (plasma gel, 7.5 mL) for chemical analysis and one Sarstedt S-Monovette (EDTA, 2.7 mL) (Sarstedt, Nümbrecht) for hematological analysis were drawn. Hematocrit was determined using Sysmex XE 2100 (Sysmex Corporation, Japan) (Imeri et al., 2008); plasma [Na^+^], plasma [K^+^] and plasma urea were determined using the Modula SWA biochemical analyzer, Modul P + ISE (Hitachi High Technologies Corporation, Japan, Roche Diagnostic) (Stockmann et al., 2008); and plasma osmolality was determined using Arkray Osmotation (Arkray Factory, Inc., Japan). Urine samples were collected in Sarstedt Urine Monovette (10 mL) and sent to the laboratory. Urine [Na^+^], urine [K^+^], and urine urea were determined using the Modula SWA biochemical analyzer, Modul P + ISE (Hitachi High Technologies Corporation, Japan, Roche Diagnostic). Urine specific gravity was determined using Au Max-4030 (Arkray Factory, Inc., Japan), and urine osmolality was determined using Arkray Osmostation OM-650 (Arkray Factory, Inc., Japan) (Zanchi et al., 2016). The K^+^/Na^+^ ratio in urine was calculated. Transtubular potassium gradient was calculated using the equation: Transtubular potassium gradient = [urine [K^+^] × plasma osmolality/(plasma [K^+^] × urine osmolality), according to West et al. (1986). Percentage change in plasma volume was determined in accordance with Van Beaumont (1972).

Blood samples are examined in clinical laboratories and strenuous exercise may have a profound effect on laboratory parameters (Kratz et al., 2002). Kratz et al. (2002) therefore provided a table of modified reference ranges for basic biochemical and hematological laboratory parameters derived from marathon runners. We compared these modified reference ranges with the values of hematocrit observed in our study. The modified reference ranges without sex difference include pre-race (39–49%) and post-race (38–48%) hematocrit values and post-race plasma osmolality values (273–318 mOsm) (Kratz et al., 2002). Plasma osmolality clinical reference range was 280–303 mOsmol/kg (Fischback and Dunning, 2004). Individual post-race urine specific gravity samples were compared with the values provided by Armstrong et al. (1998), i.e., urine specific gravity within the range of 1.013–1.029 g/mL is considered normal, values over 1.030 indicate significant dehydration and values below 1.012 g/mL indicate hyperhydration.

Anthropometric characteristics were determined in order to estimate fat-free mass, fat mass and percent body fat at B_1_ and at A_1_, A_4_, and A_7_. Body mass was measured to the nearest 0.1 kg using a commercial scale (Beurer BF 15, Beurer GmbH, Ulm, Germany). Hydration status was assessed according to the criteria proposed by Noakes et al. (2005) with overhydration classified as any weight gain above the initial body mass, euhydration as a decrease in body mass of 0.01–3.0%, and dehydration as any decrease in body mass greater than 3.0%. Skinfold measurements were taken on the right side of the body at eight sites (pectoralis, axillar, triceps, subscapular, abdomen, suprailiac, front thigh, and medial calf) using a skinfold caliper (Harpenden skinfold caliper, Baty International Ltd) and recorded to the nearest 0.2 mm. Fat-free mass (kg) was estimated using an equation for male (Stewart and Hannan, 2000) and female (Warner et al., 2004) athletes. Fat mass (kg) was calculated by subtracting fat-free mass from total body mass. Percent body fat was estimated using a specific equation for men (Ball et al., 2004a) and women (Ball et al., 2004b).

### Statistical analysis

All variables were expressed as mean and standard deviation. The data was tested for normality using the Kolmogorov-Smirnov test. Since most variables were normally distributed, parametric statistics was used for all variables to be comparable. Pearson's correlation coefficient r was used to examine relationships between the variables. The magnitude of correlation coefficients was considered trivial if *r* ≤ 0.10, small if 0.10 ≤ *r* < 0.30, moderate if 0.30 ≤ *r* < 0.50, great if 0.50 ≤ *r* < 0.70, very great if 0.70 ≤ *r* 0.90, nearly perfect if ≥ 0.90 and perfect if *r* = 1.00 (Batterham and Hopkins, 2006). A between-within subjects analysis of variance (ANOVA) examined pre- and post-race differences. The magnitude of the differences was examined with eta squared (η^2^), which was classified as small (0.010 < η^2^ ≤ 0.059), moderate (0.059 < η^2^ ≤ 0.138) and great (η^2^ > 0.138). The significance was set at alpha = 0.05. All statistical analyses were performed using MINITAB (Version 17.2; Minitab, Inc., USA) and SPSS software.

## Results

A total of 197 ultra-athletes entered the race and 53 (27%) finished all seven stages. Twenty-six runners (twenty men and six women) (87%) of the 30 who volunteered to participate in our study finished the whole race. Four athletes dropped out due to injuries or total fatigue. Pre-race training, average running speed and race time per day of each finisher are summarized in Table 2.

**Table 2:** Pre-race experience, training, running speed of the finishers and race time per stage.

Male sex (%)	76.9
Age (years)	42.6 (6.2)
Years as an active runner (years)	3.6 (1.7)
Number of finished ultra-marathons (n)	6.2 (10.3)
Mean weekly training volume in running (km)^†^	61.6 (26.1)
Longest weekly run (km)^†^	64.2 (30.7)
Average racing speed (km/h) (stage 1)	9.34 (6.55–13.60)
Average racing speed (km/h) (stage 4)	9.25 (7.47–12.78)
Average racing speed (km/h) (stage 7)	9.01 (6.53–13.16)
Average race time (h:min) per stage 1	4:43 (3:09–6:55)
Average race time (h:min) per stage 4	4:43 (3:21–5:45)
Average race time (h:min) per stage 7	4:46 (3:11–6:25)

† *During the 3 months before the event. Data are expressed as mean (SD) or mean (range)*.

### Body mass and body fat

**Figure 1:**
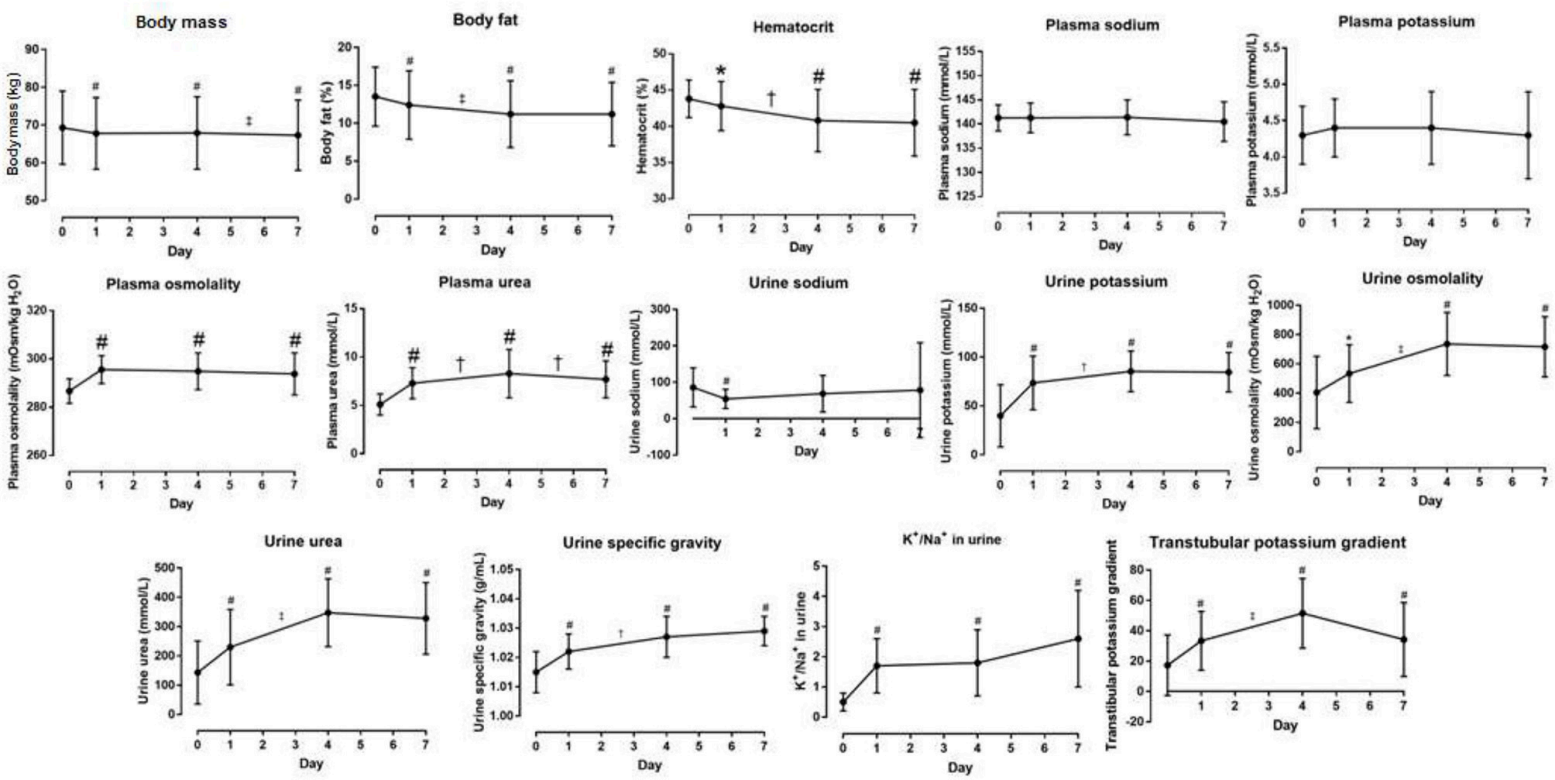
Body composition, blood and urine data and their changes. Symbol # denotes difference from day 0 = B_1_ (before stage 1) at *p* < 0.01. Symbols † and ‡ denote differences between two days at *p* < 0.05 and *p* < 0.01, respectively.

**Figure 2:**
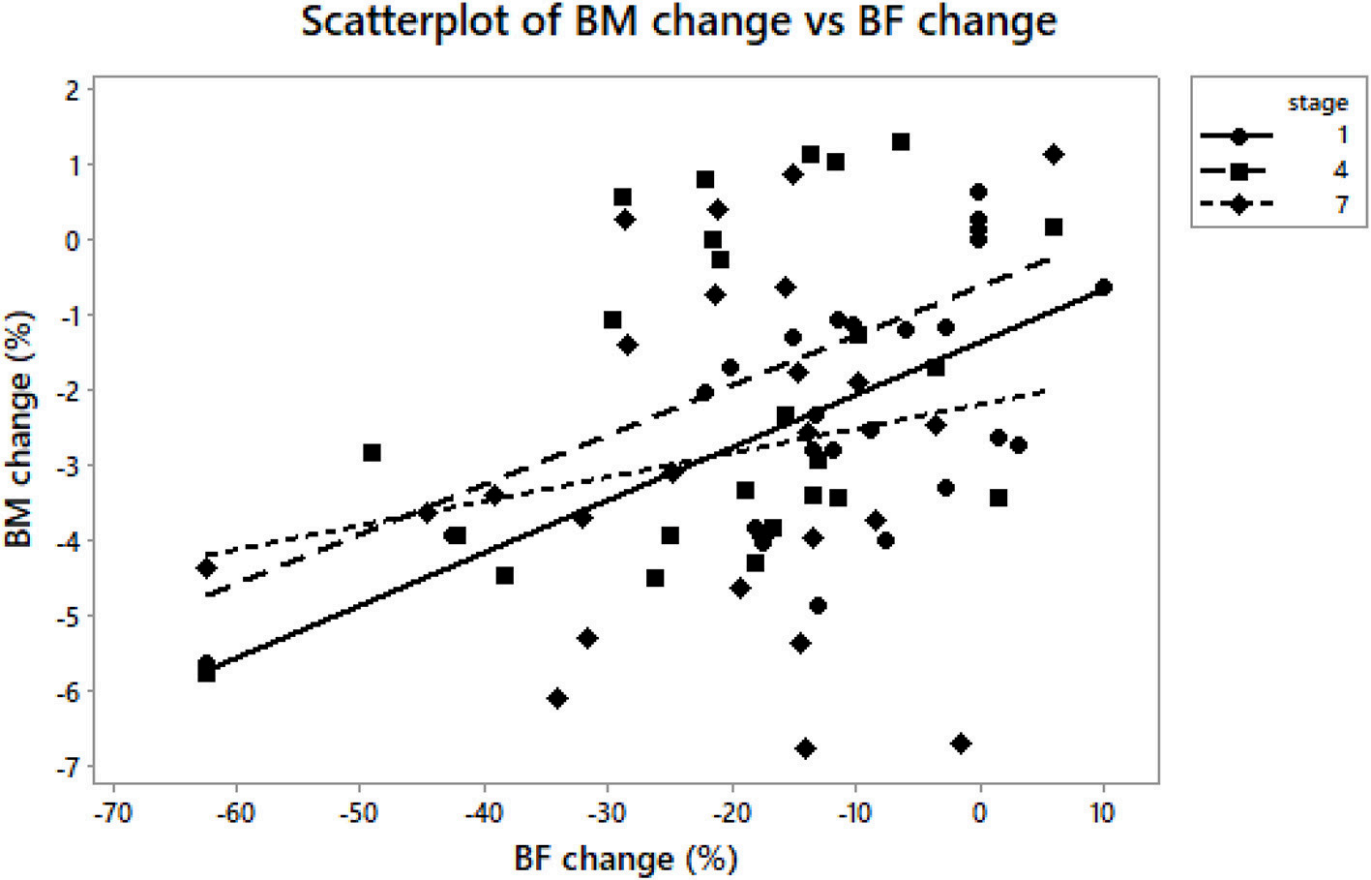
BM, body mass; BF, body fat. A scatterplot of body mass change vs. body fat change at A_1_, A_4_, and A_7_. Body mass change was associated with body fat change (*r* = 0.52, *p* = 0.007) at A_1_ vs. B_1_ (*r* = 0.42, *p* = 0.03) at A_4_ vs. B_1_ (*r* = 0.42, *p* = 0.03); at A_7_ vs. B_1_ (*p* > 0.05).

On average, body mass decreased significantly at A_1_, A_4_, A_7_ vs. B_1_ (Table 3) and A_7_ vs. A_4_ (*p* < 0.001, η^2^ = 0.501) (Figure 1). Ultra-runners were distributed by their hydration status based on body mass changes in accordance with the study by Noakes et al. (2005). There were 12% overhydrated, 61% euhydrated and 27% dehydrated athletes at A_1_; 27% overhydrated, 31% euhydrated and 42% dehydrated runners at A_4_, and 15% overhydrated, 31% euhydrated and 54% dehydrated runners at A_7_. Body fat decreased significantly at A_1_, A_4_, A_7_ vs. B_1_; A_4_ vs. A_1_ (Table 3) and A_7_ vs. A_1_ (*p* < 0.001, η^2^ = 0.572) (Figure 1). A scatterplot of percentage change in body mass vs. percentage body fat change is shown in Figure 2. Body mass change was not associated with plasma osmolality or urine specific gravity (*p* > 0.05).

**Table 3:** Results of selected body composition and blood and urine parameters of runners along multistage marathon competition.

**Parameter**	**B_1_**	**A_1_**	**Absolute change (A_1_ vs. B_1_)**	**A_4_**	**Absolute change (A_4_ vs. B_1_)**	**A_7_**	**Absolute change (A_7_ vs. B_1_)**
Body mass (kg)	69.3(9.7)	67.8(9.5)	−1.5(1.2)^**^	67.9(9.6)	−1.4(1.6)^**^	67.3(9.3)	−2.0(1.7)^**^
Body fat (%)	13.5(3.9)	12.4(4.5)	−1.1(1.2)^**^	11.2(4.4)	−2.3(1.7)^**^	11.2(4.2)	−2.3(1.5)^**^
Hematocrit (%)	43.8(2.6)	42.8(3.4)	−1.0(2.2)^*^	40.8(4.3)	−3.0(3.2)^**^	40.5(4.6)	−3.3(3.5)^**^
Plasma sodium (mmol/L)	141.3(2.7)	141.3(3.1)	0.0(2.9)	141.4(3.6)	0.1(3.2)	140.5(4.1)	−0.8(4.1)
Plasma potassium (mmol/L)	4.3(0.4)	4.4(0.4)	0.1(0.4)	4.4(0.5)	0.1(0.5)	4.3(0.6)	0.0(0.7)
Plasma osmolality (mOsm/kg H_2_O)	286.7(5.1)	295.6(5.8)	8.9(7.2)^**^	294.9(7.6)	8.2(7.1)^**^	293.8(8.7)	7.1(8.7)^**^
Plasma urea (mmol/L)	5.1(1.1)	7.3(1.6)	2.2(1.2)^**^	8.3(2.5)	3.2(2.3)^**^	7.7(1.9)	2.6(1.8)^**^
Urine sodium (mmol/L)	85.6(53.9)	54.1(26.3)	−31.5(53.6)^**^	68.8(50.4)	−16.8(81.1)	78.2(130.4)	−7.4(139.9)
Urine potassium (mmol/L)	39.9(31.8)	73.6(27.6)	33.7(35.0)^**^	85.3(20.9)	45.4(31.7)^**^	84.5(20.2)	44.6(32.8)^**^
Urine osmolality (mOsm/kg H_2_O)	404.5(246.3)	534.0(196.6)	129.5(291.1)^*^	735.6 (214.5)	331.1(252.9)^**^	715.9(206.2)	311.4(273.4)^**^
Urine urea (mmol/L)	143.5(107.7)	229.7(128.9)	86.2(110.2)^**^	347.3(115.7)	203.8(115.0)^**^	327.9(122.4)	184.4(144.9)^**^
Urine specific gravity (g/mL)	1.015(0.007)	1.022(0.006)	0.007(0.009)^**^	1.027 (0.007)	0.012(0.008)^**^	1.029(0.005)	0.014(0.010)^**^
K^+^/Na^+^ ratio in urine	0.5(0.3)	1.7(0.9)	1.2(0.9)^**^	1.8(1.1)	1.3(1.2)^**^	2.6(1.6)	2.1(1.6)^**^
Transtubular potassium gradient	17.3(19.9)	33.4(19.4)	16.1 (25.5)^**^	51.5(23.0)	34.2(24.4)^**^	50.2(22.6)	32.9(27.7)^**^

*Mean (SD)*.

* p ≤ 0.05 and

** *p < 0.01 changes after stage 1, 4, and 7 vs. before stage 1. B_1_, before stage 1; A_1_, after stage 1; A_4_, after stage 4; A_7_, after stage 7*.

### Plasma [Na^+^], plasma [K^+^], reported fluid intake

No change was observed in plasma [Na^+^] (*p* = 0.326, η^2^ = 0.044) and [K^+^] **(***p* = 0.900, η^2^ = 0.005) (Table 3, Figure 1). Plasma [Na^+^] of < 135 mmol/L was found in one male athlete who finished the competition with plasma [Na^+^] of 135 mmol/L (A_1_), 134 mmol/L (A_4_) and 130 mmol/L (A_7_). However, this athlete was already hyponatremic at pre-race testing (132 mmol/L) and plasma [Na^+^] concentration was higher after two of the three stages.

**Figure 3:**
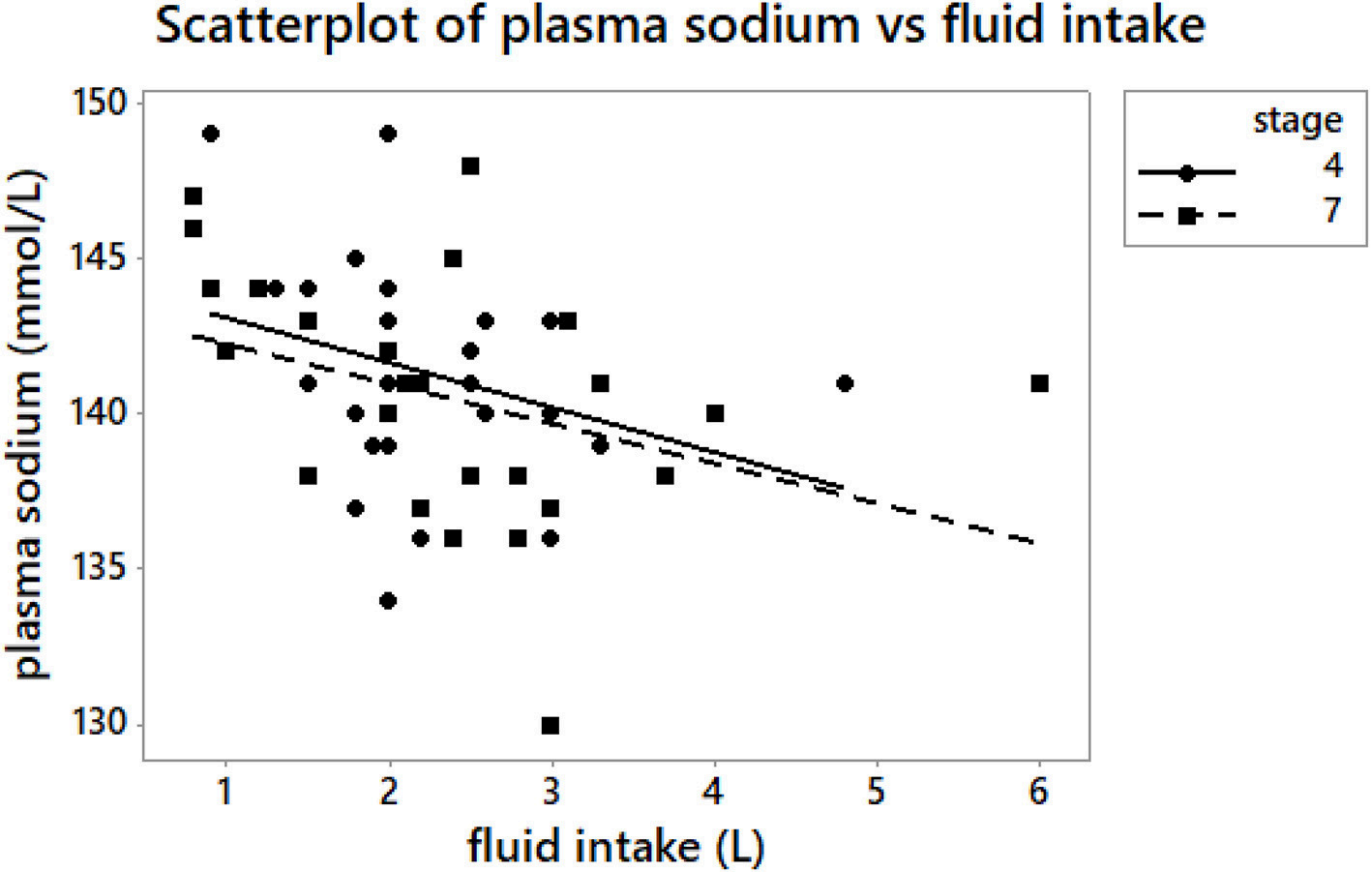
A scatterplot of plasma [Na^+^] vs. reported fluid intake at A_4_ and A_7_. Fluid intake – fluid intake during the stage. Fluid intake during stage 4 was negatively correlated with plasma [Na^+^] (*r* = −0.42, *p* = 0.007) at A_4_. Fluid intake during stage 7 was negatively associated with plasma [Na^+^] (*r* = −0.50, *p* = 0.009) at A_7_.

The average reported fluid intake during stages 1, 4, and 7 was 0.5 (0.4) L/h. That is 6.1 (2.2) mL/kg/h during stage 1; 7.0 (2.6) mL/kg/h during stage 4, and 7.5 (3.2) mL/kg/h during stage 7. Fluid intake during stage 4 was negatively correlated with plasma [Na^+^] (*r* = −0.42, *p* = 0.007) (Figure 3) and plasma [K^+^] (*r* = −0.43, *p* = 0.03) at A_4_. Fluid intake during stage 7 was negatively associated with plasma [Na^+^] (*r* = −0.50, *p* = 0.009) (Figure 3) and plasma osmolality (*r* = −0.53, *p* = 0.005) at A_7_. Fluid intake during the stages was not associated with body mass or percentage plasma volume change at A_1_, A_4_, and A_7_ (*p* > 0.05).

### Plasma osmolality, plasma urea

**Figure 4:**
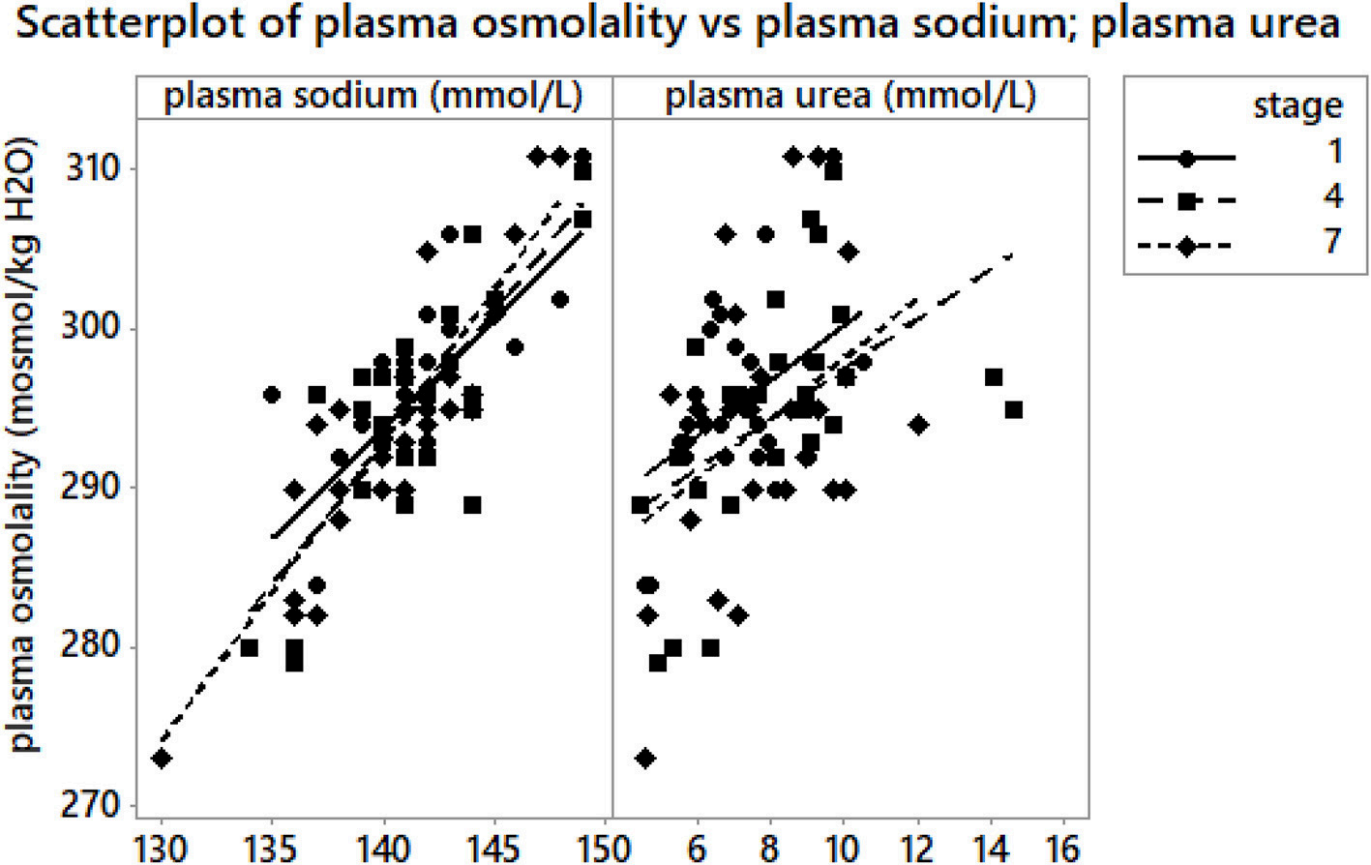
A scatterplot of plasma osmolality vs. plasma [Na^+^] and plasma urea at A_1_, A_4_, and A_7_. Plasma osmolality was associated with plasma [Na^+^] (*r* = 0.69, *p* < 0.001) at A_1_, (*r* = 0.64, *p* < 0.001) at A_4_ and (*r* = 0.42, *p* = 0.03) at A_7_. Plasma osmolality was correlated with plasma urea at A_1_ (*r* = 0.59, *p* = 0.001) and at A_4_ (*r* = 0.58, *p* = 0.002); at A_7_ (*p* > 0.05).

Plasma osmolality was within the modified reference range for marathoners proposed by Kratz et al. (2002). Plasma osmolality significantly increased at A_1_ vs. B_1_, A_4_ vs. B1 and A_7_ vs. B_1_ (*p* < 0.001, η^2^ = 0.416) (Table 3); but remained stable at A_4_ vs. A_1_, A_7_ vs. A_4_, A_7_ vs. A_1_. Plasma urea significantly increased at A_1_ vs. B_1_, A_4_ vs. B_1_ and A_7_ vs. B_1_ (*p* < 0.001) (Table 3), A_4_ vs. A_1_ (*p* = 0.01) and A_7_ vs. A_4_ (*p* < 0.001, η^2^ = 0.586). Absolute plasma osmolality and plasma urea changes are shown in Figure 1. A scatterplot of plasma osmolality vs. plasma [Na^+^] and plasma urea is detailed in Figure 4.

### Hematocrit, plasma volume

Pre-race hematocrit values ranged from 38.5 to 49%, and were within the modified reference ranges established for marathon runners (Kratz et al., 2002). Post-race hematocrit values (38–48%) were achieved by all the finishers at A_1_, whereas 12% of the athletes (2 men and 1 woman) showed lower values at A_4_, and 12% (1 man and 2 women) at A_7_, i.e., they were anemic (Kratz et al., 2002). Hematocrit significantly decreased at A_1_ vs. B_1_, A_4_ vs. B_1_, A_7_ vs. B_1_ (Table 3), A_4_ vs. A_1_ and at A_7_ vs. A_1_ (*p* < 0.001, η^2^ = 0.358) (Figure 1). Percentage change in plasma volume was 2.1(7.7)% at A_1_, 12.4(11.1)% at A_4_ and 13.3(13.7)% at A_7_, calculated according to Van Beaumont (1972). Percentage change in plasma volume at A_4_ and A_7_ was significantly higher than the value at A_1_ (*p* = 0.001, *p* = 0.002, respectively). Percentage change in plasma volume was not correlated with plasma or urine osmolality change (*p* > 0.05).

### Urine [Na^+^] and [K^+^], K^+^/Na^+^ ratio in urine, transtubular potassium gradient

There was no change in urine [Na^+^] (*p* = 0.386, η^2^ = 0.036) (Table 3, Figure 1). Urine [K^+^] increased at A_1_, A_4_, A_7_ vs. B_1_ (Table 3), and at A_7_ vs. A_1_ (*p* < 0.001, η^2^ = 0.507) (Figure 1). The K^+^/Na^+^ ratio in urine significantly increased at A_1_, A_4_, A_7_ vs. B_1_ (*p* < 0.001, η^2^ = 0.430) (Table 3, Figure 1). Transtubular potassium gradient significantly increased at A_1_ vs. B_1_, A_4_, A_7_ vs. B_1_ (Table 3), A_4_ vs. A_1_ and A_7_ vs. A_1_ (*p* < 0.001, η^2^ = 0.560) (Figure 1).

### Urine osmolality, urine urea, urine specific gravity

**Figure 5:**
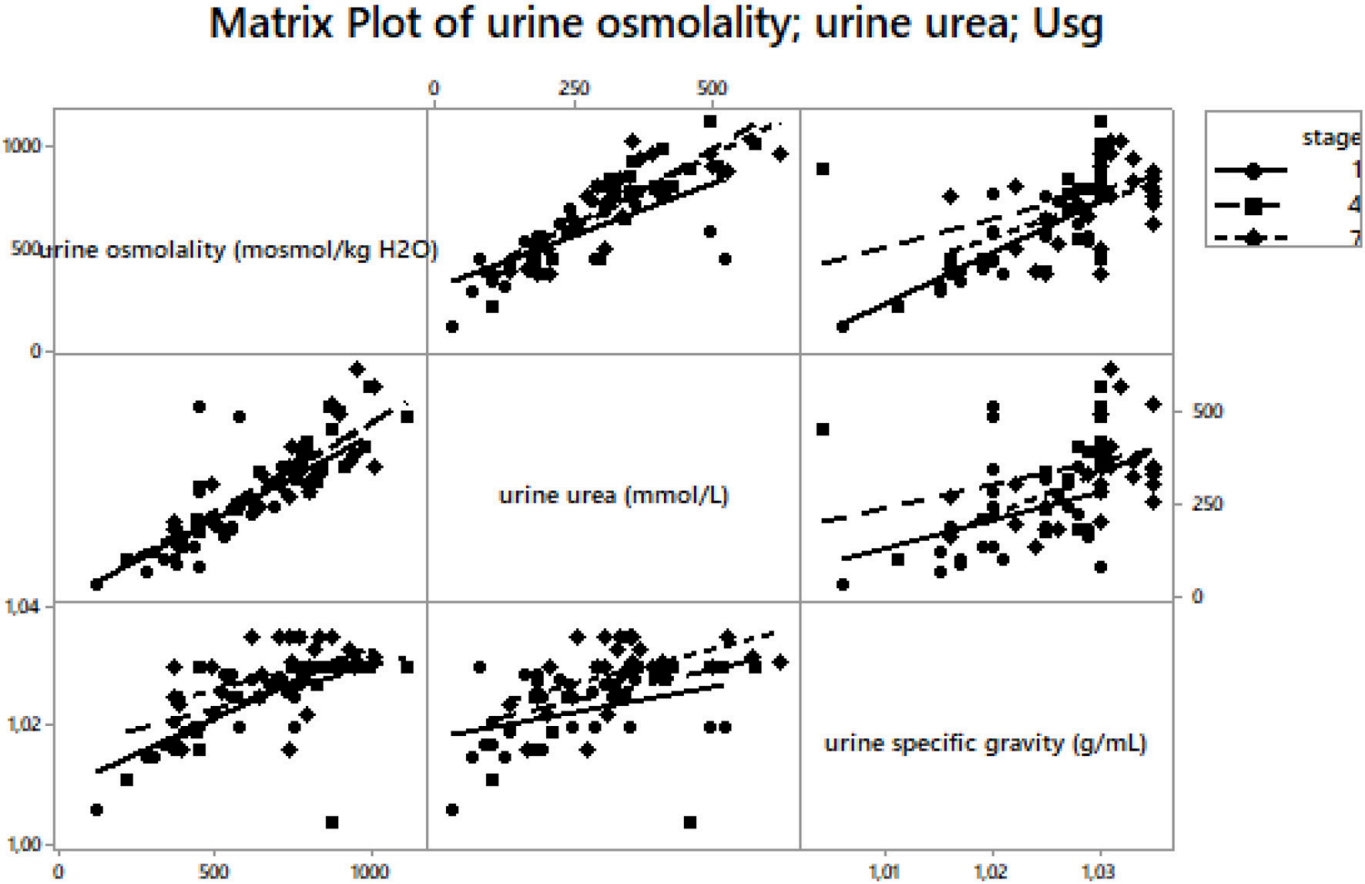
A matrix plot of urine osmolality, urine urea and urine specific gravity at A_1_, A_4_ and A_7_. Usg – urine specific gravity. Urine osmolality was significantly associated with urine specific gravity (*r* = 0.71, *p* < 0.001) at A_1_, (*r* = 0.52, *p* = 0.006) at A_4_ and (*r* = 0.46, *p* = 0.02) at A_7_. Urine osmolality was significantly associated with urine urea at A_1_ (*r* = 0.80, *p* < 0.001), at A_4_ (*r* = 0.81, *p* < 0.001) and at A_7_ (*r* = 0.86, *p* < 0.001). Urine specific gravity was associated with urine urea (*r* = 0.39, *p* = 0.05) at A_1_. Other correlations were not significant (*p* > 0.05).

Urine osmolality significantly increased at A_1_ vs. B_1_; A_4_ vs. B_1_, A_7_ vs. B_1_ (Table 3), A_4_ vs. A_1_ and A_7_ vs. A_1_ (*p* < 0.001, η^2^ = 0.465) (Figure 1). Urine urea significantly increased at A_1_ vs. B_1_, A_4_, A_7_ vs. B_1_ (Table 3), A_4_ vs. A_1_ and A_7_ vs. A_1_ (*p* < 0.001, η^2^ = 0.532) (Figure 1). Urine specific gravity significantly increased at A_1_, A_4_, A_7_ vs. B_1_ (Table 3), A_7_ vs. A_1_ (*p* < 0.001, η^2^ = 0.540) (Table 3, Figure 1). There were 4% hyperhydrated and 12% dehydrated athletes at A_1_; 4% hyperhydrated and 50% dehydrated at A_4_, and 0% hyperhydrated and 65% dehydrated at A_7_ based on the athletes' individual post-race urine specific gravity samples. A matrix plot of urine osmolality, urine specific gravity and urine urea at A_1_, A_4_, and A_7_ is detailed in Figure 5.

## Discussion

We investigated the effect of a multi-stage race in which athletes have to run one marathon per day on the parameters of fluid metabolism. Running one marathon per day led to a body mass decrease without any disturbance of fluid homeostasis. Plasma sodium, plasma potassium and urine sodium remained stable at pre-race (baseline) levels at A_1_, A_4_, and A_7_ vs. B_1_. We have confirmed the hypothesis that body mass changes do not reflect changes in the hydration status of runners. Also of note is the finding of a negative relationship between the reported fluid intake and plasma sodium at A_4_ and A_7_.

### Body mass and body fat decrease

The average body mass loss was 2.9%, which is relatively low compared to the studies conducted by Zouhal et al. (2009) during a 7-day, self-sufficient, multi-stage running race across the desert, and Hue et al. (2014) during a 6-day, 142-km trail running race, both in hot environments (~30 or ~25°C, respectively). By contrast, body mass loss was higher than in the 5-day, self-sufficient multi-stage ultramarathon in a hot ambient environment (~36°C) in the study by Costa et al. (2013), during the longest 7-day multi-stage mountain ultramarathon in Europe called the “Swiss Jura Marathon” with temperatures ranging from 8 to 14°C in the study by Knechtle et al. (2010a, 2011, 2012a), and during a 5-day multi-stage ultra-endurance run where athletes had to run 338 km in 5 days with temperatures ranging from 19 to 22°C (Knechtle and Kohler, 2007). The strenuous conditions of a 7-day, multi-stage marathon run also led to a significant decrease in body fat. A decrease in body mass is often the consequence of body fat decrease (Helge et al., 2003; Knechtle et al., 2005; Bircher et al., 2006). A decrease in fat mass is expected and the ability to use body fat as fuel is important in such ultra-endurance athletes (Raschka and Plath, 1992). When considering body mass and body fat changes, it should be recognized that, in contrast to continuous events, the athletes had time between the stages for fluid and nutritional restoration. Therefore, although there may be some minor cumulative mass loss, the main loss of mass probably occurred during each stage. The decrease in body mass and the increase in urine specific gravity after each observed stage in comparison with pre-race values might also be due to dehydration (Armstrong et al., 1998). Although sweat rate was not assessed, the temperature rose gradually from stage to stage (~24°C) and increased by 12 degrees during the seventh stage. The highest number of dehydrated athletes based on their body mass changes (Noakes et al., 2005) was found at A_7_. A similar number of athletes were probably dehydrated after a multi-stage ultramarathon in the study of Krabak et al. (2017). Nevertheless, the present runners had a significantly higher percentage change in plasma volume than after A_1_ and no relationship between the change in plasma volume and plasma osmolality or fluid intake was found. Moreover, body mass changes were not associated with urine specific gravity changes in these ultramarathoners and the loss of body mass is an imperfect predictor of total body volume (Hew-Butler et al., 2015). A recent study of a 161-km ultramarathon by Hoffman and Stuempfle (2016) defines dehydration as a decrease in body mass greater than 4%, which would mean that in our study, 15% of the athletes were dehydrated at A_1_, 15% at A_4_ and 27% at A_7_–much less than with the original calculations defined by Noakes et al. (2005). The number of dehydrated athletes might vary depending on the definition of dehydration based on race distance (Noakes et al., 2005; Hoffman and Stuempfle, 2016). It has been estimated that ~3–4% body mass should be lost during prolonged exercise in order to maintain euhydration (Noakes et al., 2005; Hoffman and Stuempfle, 2014) and 1% of body mass loss could be due to fat utilization in prolonged races (Stuempfle et al., 2011). During prolonged exercise, body mass loss does not exactly reflect body water loss, due to the effects of change in body mass by the release of water bound with muscle and liver glycogen, substrate use and production of water during substrate metabolism (Hoffman et al., 2017). Notwithstanding, we found a positive relationship between urine specific gravity changes and urine urea changes, most likely as a result of increased protein catabolism (Noakes and Carter, 1976; Warburton et al., 2002). During endurance activities, the body primarily aims at maintaining sodium and plasma osmolality, but not body mass (Tam et al., 2011).

### Plasma sodium, plasma potassium, reported fluid intake

Overall, the ultramarathoners in our study maintained plasma sodium and potassium levels. Blood sodium levels are not affected by sodium losses during exercise, but by the associated changes in body water content (Noakes, 2012). Sodium and potassium remained within the normal range, similarly as after a 142-km trail running race in tropical conditions (~30°C) (Hue et al., 2014). Post-race serum sodium concentrations did not differ among the five stages of a 225-km multi-stage ultramarathon (~36°C) (Costa et al., 2013). Similarly, plasma sodium and potassium remained unchanged during a 7-day multi-stage mountain ultramarathon (~17°C) (Knechtle et al., 2011, 2012a).

We would expect an association between the reported fluid intake and post-race plasma sodium, and indeed, runners with a lower fluid intake showed higher plasma sodium levels at A_4_ and A_7_. Nevertheless, the runners in current study showed no occurrence of EAH. We have to take into consideration the fact that the reported amount of fluids could be under- or overestimated by the athletes or assistants at fluid stations. In any case, the reported average intake of 0.5 L/h was probably appropriate to maintain body water despite the body mass loss.

### Plasma osmolality, plasma urea

Plasma osmolality increased similarly as in the studies by Knechtle et al. (2012a) or Costa et al. (2013) and was related to plasma sodium. Aldosterone increases sodium reabsorption and raises plasma osmolality by increasing the excretion of potassium from the body; increased plasma osmolality stimulates ADH production until plasma osmolality is restored to normal levels (Noakes, 2012). Plasma osmolality was within the normal clinical reference range (Fischback and Dunning, 2004) in all the ultramarathoners before the start and at A_1_, A_4_ and A_7_, except for three male runners at A_7_ (above normal clinical range) and one male finisher with plasma osmolality level below the normal clinical range pre-race and at A_7._ Moreover, plasma osmolality was not related to percentage change in plasma volume and plasma sodium remained stable post-race. Appropriate hydration probably preserved plasma osmolality within the normal range and maintained intracellular volume and homeostasis in the present multi-stage marathon run. By contrast, plasma osmolality was associated with plasma urea at A_1_ and A_4_ in similarity to the studies by Knechtle et al. (2011, 2012a). An increase in plasma urea suggests increased metabolic activity; however, it can also indicate decreased renal functions (Rama et al., 1994; Knechtle et al., 2012a,b) attributable to skeletal muscle damage and protein catabolism. An increase in plasma urea concentration (+52%) provides indirect evidence of the breakdown of proteins in the body (Dressendorfer and Wade, 1991). Higher post-race plasma urea occurs in a high percentage of ultramarathon runners who most likely finish faster and have greater body mass losses (Hoffman and Weiss, 2016). We assume that the increase in plasma urea concentrations induced the increase in plasma osmolality.

### Hematocrit, plasma volume

The decrease in hematocrit was similar to that observed in the literature on multi-stage endurance ultra-running races (Raschka and Plath, 1992; Zouhal et al., 2009; Knechtle et al., 2010a, 2011, 2012a; Rüst et al., 2012; Rama et al., 2016). Hemodilution might be caused by sodium retention and potassium secretion due to increased aldosterone activity, as well as due to the increased secretion of ADH with fluid conservation (Dressendorfer and Wade, 1991; Noakes, 2012). An increase in plasma volume with hemodilution and decreased hematocrit is often observed after prolonged exercise of moderate intensity over several consecutive days (Fellmann et al., 1999; Knechtle et al., 2010a, 2011, 2012a; Costa et al., 2013). During prolonged exercise, plasma volume tends to remain constant or increase due to the movement of water from the intracellular to the extracellular compartment (Fellmann et al., 1999; Zouhal et al., 2009). Plasma volume increase is also represented by the production of metabolic water from the oxidation of fatty acids and carbohydrates and glycogen degradation in muscles and in the liver (Pastene et al., 1996). It is necessary to mention that the missing correction of blood parameters prior to stage 4 and 7 according to the change of plasma volume throughout the race week may have confounded the results and the conclusions of the study. In any case, considering “pseudoanemia” as a response to prolonged strenuous endurance exercise as well as the possibility of intravascular hemolysis from mechanical trauma and oxidative injury of red blood cells reflects the impact of physical exertion of an ultramarathon (Rama et al., 2016).

### Urine osmolality, urine specific gravity, urine urea

Urine osmolality increased and was associated with increased urine specific gravity which was related to increased urine urea. Increased urine osmolality was also reported by Costa et al. (2013) during and after a five-stage 225 km ultramarathon and by Knechtle et al. (2012a) after a 7-day multi-stage ultramarathon. Increased urine osmolality and its high levels are a common feature of exercise-heat stress and can indicate a state of water conservation (Armstrong, 2007). By contrast, it seems that urine osmolality did not reflect body water content during a multi-stage ultra-race in the heat reported by Costa et al. (2013). Runners in their study had urine osmolality above the reference range and showed an increased plasma volume, just as the runners in the present study. Similarly, percentage plasma volume change was not associated with urine osmolality change in the present ultramarathoners. According to Costa et al. (2013), urine measure of hydration is insufficient in monitoring hydration status. Catabolic products of protein metabolism could also lead to increased urine osmolality, which limits its potential usefulness for the assessment of dehydration (Cheuvront et al., 2013). An increase in urine urea may be related to increased protein catabolism, secondary to the reduction in renal blood flow, all of which may occur after prolonged strenuous exercise (Noakes and Carter, 1976).

### Potassium/sodium ratio in urine, transtubular potassium gradient

The potassium/sodium (K^+^/Na^+^) ratio in urine was < 1 before B_1_, increased and was > 1 at A_1_, A_4_, and A_7_ in the present study. Sodium and potassium concentrations are indirect markers of aldosterone and ADH secretion (Knechtle et al., 2012a). The K^+^/Na^+^ ratio in urine was < 1 before the multi-stage ultra-endurance race and was > 1 after each stage in the case study by Knechtle et al. (2010a). The K^+^/Na^+^ ratio also increased in the study focusing on multi-stage ultra-endurance triathletes (Knechtle et al., 2012b). The limitation was that we did not observe the K^+^/Na^+^ ratio in urine at B_4_ and B_7_. Nevertheless, the urine K^+^/Na^+^ ratio > 1 may reflect contraction of the extracellular volume leading to hyperreninemic hyperaldosteronemia and could be interpreted as a reaction to the stimulation of the renin-angiotensin-aldosterone-system (RAAS) (Wade et al., 1981); however, we did not measure the RAAS. Transtubular potassium gradient also increased and reached values > 10, indicating an increased aldosterone activity (Gault et al., 1992). It is important to note that there is a limitation to the transtubular potassium gradient calculation, as it requires urine sodium > 25 mmol/L (Choi and Ziyadehm, 2008) so that sodium delivery to the cells is not rate-limiting for potassium secretion. 15% of the present runners did not reach this level at A_1_ and A_4_ and 35% at A_7_. Nevertheless, the present findings suggest that a positive K^+^/Na^+^ ratio in urine was influenced by increased aldosterone activity and more potassium than sodium was excreted by the kidneys during the stages. Increased aldosterone is a physiological reaction in multi-stage ultra-runners according to Wade et al. (1981). The RAAS increases transtubular potassium ratio during the race to preserve plasma sodium. This may also explain why plasma sodium remained stable at the finish of the multi-stage marathon despite body mass losses.

### Limitations

This study has a few limitations worth noting. Water intake was calculated based on information reported by the competitors and assistants at fluid stations; it was not analyzed through dietary analysis software and water in solid food was not recorded. The absence of data on renal hormone responses, urine losses, metabolic water gain, respiratory water and sweat losses may limit the present study. Another limitation is the missing differentiation between sexes that are considered of importance regarding the risk profile for exercise associated dysnatremia. Notwithstanding, we believe our findings provide a foundation for future studies because our methodology is the same as previous well-established studies of marathon and single-stage races (Noakes et al., 2005; Hoffman et al., 2013b) and multi-stage races (Krabak et al., 2017) although they investigated larger numbers of participants. Finally, limited blood and urine samplings only on day 4 and 7 could mask some potential relationships. The main reason noted were a lack of time to collect and process the blood and urine samples prior to all stages, the sake of the athletes' comfort and the runners' inability to prepare for the start in time. After the stage at the finish the participants were more able and willing to take part in laboratory measurements.

### Practical applications

The findings of the present study support the hypothesis that body mass changes do not reflect changes in the hydration status during prolonged exercise of several hours. As hydration status is a major concern for ultra-endurance runners and professionals working with them, future hydration guidelines should consider these findings in order to help coaches, trainers, sport nutritionists and doctors develop tailored nutritional strategies during a race.

## Conclusions

Fluid and electrolyte balance was maintained without any decrease in plasma volume at the finish of a 7-day multi-stage marathon run. We presume that the decrease in body mass could be the result of the metabolic breakdown of fuel, which includes body fat loss, endogenous substrate use, sweat loss, respiratory and urinary losses, and/or fecal body mass loss during running. The lack of change in body fluid balance may be due to the distance of a marathon.

## Author contributions

DC: designed the study, collected all data and drafted the manuscript, PN, TR, and BK helped in designing the study and drafting the manuscript, JB performed the statistical analyses.

### Conflict of interest statement

The authors declare that the research was conducted in the absence of any commercial or financial relationships that could be construed as a potential conflict of interest.
